# The influence of sociodemographic factors and response style on caregiver report of infant developmental status

**DOI:** 10.3389/fped.2022.1080163

**Published:** 2023-01-11

**Authors:** Amy K. Connery, Radhika S. Raghunathan, Alison M. Colbert, Laszlo Erdodi, Seth Warschausky, Alissa Huth-Bocks, H. Gerry Taylor, Trivellore Raghunathan, Patricia Berglund, Angela D. Staples, Angela Lukomski, Jazmine Kirkland, Jennifer Cano, Renee Lajiness-O’Neill, the PediaTrac Project Consortium**

**Affiliations:** ^1^ Children’s Hospital Colorado, Department of Physical Medicine and Rehabilitation, University of Colorado School of Medicine, Aurora, CO, United States; ^2^ Department of Mental Health, Johns Hopkins Bloomberg School of Public Health, Baltimore, MD, United States; ^3^ Psychology, University of Windsor, Ontario, ON, Canada; ^4^ Physical Medicine and Rehabilitation, Michigan Medicine, Ann Arbor, MI, United States; ^5^ Pediatrics, Case Western Reserve University, University Hospitals, Cleveland, OH, United States; ^6^ Abigail Wexner Research Institute at Nationwide Children’s Hospital, and Pediatrics, The Ohio State University, Columbus, OH, United States; ^7^ Institute of Social Research, University of Michigan, Ann Arbor, MI, United States; ^8^ Psychology, Eastern Michigan University, Ypsilanti, MI, United States; ^9^ Nursing, Eastern Michigan University, Ypsilanti, MI, United States

**Keywords:** caregiver report, infant development, early childhood development, response style, sociodemographic factors

## Abstract

Caregiver report is the most feasible way to assess early childhood development but is susceptible to the influences of response style and sociodemographic factors. In a sample of 571 caregiver-infant dyads (47.8% female; 48% White), we compared caregiver reports on the Ages and Stages Questionnaire-Third Edition (ASQ-3) with reports on a novel, web-based assessment, PediaTrac™. Ratings on PediaTrac correlated with ratings on the ASQ-3 at all time points (2, 4, 6, and 9 months). Caregiver age, response style, and sociodemographic factors accounted for significant variance on both measures. Developmental reporting of early childhood skills is influenced by caregiver response style and sociodemographic factors. These influences must be considered in order to ensure the accurate identification of infant developmental status.

## 1. Introduction

While there is widespread consensus that the identification of and early intervention for childhood developmental delays can improve outcomes and prevent long-term problems (1, 2), there are limited reliable and valid methods available for detecting these delays within the first two years of life (3). Reliable methods of assessing children during infancy and early toddlerhood are needed to improve developmental surveillance and better target interventions (4, 5).

Tests of early developmental skills with strong psychometric properties do exist (6, 7). While these performance-based tests are often considered to be the “gold standard,” they are costly, time and resource-intensive and require specialized personnel for administration and interpretation (8). Typically, these tests are not feasible in the context of well child medical visits, routine developmental monitoring programs, and large-scale research projects. Moreover, they are often only available to children already identified as “at risk” and to children living in more urban, or highly resourced areas.

A more feasible method of developmental monitoring and assessment is caregiver (e.g., parent) report. Caregiver report is commonly used in pediatric well child visits and developmental outcome research (4, 5). Research outcomes and conclusions, as well as decisions by primary care providers to refer children for further assessment or interventions, are frequently based on caregiver reports and governmental and educational institutions utilize these types of metrics for financial and programming decisions (9). However, these tools are not without limitations, and these shortcomings are frequently underestimated, underappreciated or not considered in clinical and policy decision making and research, leading to increased measurement error at the individual level and group misassignment in research designs (8).

While caregiver reports can assess a wide spectrum of child behaviors observed in daily life by adults who spend substantial time with the child, these reports can be subject to bias. Factors contributing to bias include differing levels of caregiver knowledge about typical and atypical development (9, 10), concerns about stigmatization (11, 12) and cultural differences in what constitutes normal and abnormal development (12–14). High levels of caregiver stress can also influence reports of a child's developmental status (15, 16). Some caregiver report measures available for children over 2 years of age include embedded measures of response style (17, 18). However, these metrics are not routinely available and, to our knowledge, have not been included in any caregiver report of infant and toddler development (6, 19, 20).

In addition to response style, caregiver reports are also influenced by sociodemographic factors. While one might anticipate that children with less socioeconomic advantage [e.g., lower levels of caregiver education or other indices of socioeconomic status (SES)] would receive less favorable developmental ratings than those from more advantaged backgrounds, findings from several studies fail to confirm this expectation. Two studies found that caregivers with lower levels of education tended to rate their children higher on a developmental language inventory than those with higher levels of education (21, 22). Several other studies have also shown that caregivers with lower SES may tend to rate their child's abilities more favorably than those from higher SES backgrounds (23–27). These findings are theorized to be due to concern about the child “failing” the measure, avoidance of stigmatization, differing levels of knowledge about early development or developmental expectations, or cultural differences in interpretation of item content. Despite these findings, there is no early developmental assessment tool that is capable of systematically accounting for sociodemographic factors in score interpretation.

Some researchers have suggested that caregiver report is not sufficiently sensitive or specific to early signs of delayed development to justify its widespread use (8, 28, 29); however, a recent investigation suggests that caregivers of term and preterm infants can report newborn motor abilities with high reliability (30). Data that are inaccurate can lead to diagnostic errors, the over- or under-identification of children in need, or misleading data for financial or policy programming. However, as caregiver report is likely the most feasible, cost and time efficient way to identify children early, as well as the vital nature of early identification and intervention, further efforts are warranted to improve the accuracy of this method of early developmental assessment.

PediaTrac™, is a multi-dimensional, online survey tool constructed with contemporary item response theory (IRT) modeling methods to monitor and track infant and toddler development (30). PediaTrac queries caregivers about their child's development at multiple time points, measures caregiver response style, and gathers additional information known to influence caregiver report, including sociodemographic factors.

The current study focused on assessment of early social, communication, and cognitive skills. These domains were selected based on their greater sensitivity to caregiver characteristics and response style. Specifically, research has shown that caregiver report of language and cognitive functioning may be more subjective than more directly observable domains, such as motor skills (31–33).

In order to examine the impact of response style and sociodemographic characteristics on caregiver report, we first attempted to establish that PediaTrac, a recently developed measure, was measuring early childhood developmental constructs in the same manner as another established and widely used caregiver report measure. To establish convergent validity, we compared the PediaTrac Social/Communication/Cognition domain (SCG) to comparable scales of the Ages and Stages Questionnaire-Third Edition (ASQ-3; Personal-Social, Communication, and Problem Solving). We hypothesized that the convergent validity of the PediaTrac SCG domain would be demonstrated by its association with caregiver ratings on the three ASQ-3 scales. We then hypothesized that significant variance in caregiver report on both measures would be accounted for by sociodemographic factors (i.e., maternal age and education level) and caregiver response style.

### 2. Methods

### 2.1. Participants

This investigation is part of the larger PediaTrac study, a prospective, longitudinal investigation of a sample of 571 caregivers of infants (48% female) who were born either at term (*n *= 331; 49% female) or preterm (*n *= 240; 46% female) (30). The sample was recruited from three sites that included academic medical centers and a local community clinic: 100 from Site #1, 239 from Site #2, and 232 from Site #3. Site #1 did not have access to a preterm sample and recruited only term caregiver/infant dyads from an urban academic medical center and a socio-demographically high-risk community clinic to ensure representation of systematically excluded communities in our larger term sample. Sites #2 and #3 were large academic medical centers from which both term and preterm infants were recruited, the latter from which also a socio-demographically high-risk population was recruited. Term infants had a gestational age (GA) of ≥37 weeks at birth and a minimum birth weight of 2500 g, with no history of prenatal or intrapartum complications, neonatal abstinence syndrome, neurological injury/disease, or known genetic disorder. Preterm infants had a GA of <37 weeks. Birth weight was allowed to vary, but exclusions from the preterm group included neonatal abstinence syndrome and Down syndrome. A single infant from each multiple birth was randomly selected for enrollment. Caregivers were a minimum of 18 years old and had access to a personal device such as a smartphone, tablet, or computer. Ninety-eight percent of the respondents were biological mothers. English-language proficiency was required for participation. All American Psychological Association (APA) ethical guidelines were followed and Institutional Review Board (IRB) approval was obtained.

### 2.2. Study procedures

Caregivers were recruited in their last trimester of pregnancy, after their infants' birth in the hospital, or at their first newborn visit, with consent obtained after birth. Primary caregivers of term infants completed PediaTrac soon after birth, whereas caregivers of preterm infants completed it when their infants reached a postmenstrual age of 39 weeks. Sampling periods were thus based on the corrected age for preterm infants.

### 2.3. Study measure and variables

PediaTrac v3.0 is a web-based survey comprised of between 511 and 558 unique items covering the age range from birth to 18 months (30). Information describing the original item bank and domain development, expert panel reviews, interviews with caregivers, and the pilot validation results of PediaTrac 2.0 have been previously published (34). In PediaTrac v3.0, caregivers complete subsets of the survey ranging from ∼220–340 items, depending on the sampling period of the assessment. PediaTrac queries multiple developmental domains, including Feeding/Eating/Elimination, Sleep, Motor, Social/Communication/Cognition (SCG), Early Relational Health and Social/Sensory Information Processing, at each of 8 sampling periods [newborn (NB) and 2, 4, 6, 9, 12, 15, and 18 months].

PediaTrac has been developed using item response theory (IRT) methodology, which is a measurement framework that uses mathematical models to explain the relationship between latent traits (attributes) and their observed outcome. IRT models the likelihood of a given response to an item as a probabilistic function of the individual's score on a latent trait of interest, referred to as theta (35). IRT offers benefits over classical test theory (CTT) including sample-invariant parameter estimates (i.e., assuming no differential item functioning across populations) to metrics of reliability at both the item and test level (36–38). It is an item-oriented rather than a test-oriented test construction method. As such, it lends itself to an individualized medicine approach in assessment and subsequent care.

The focus of this investigation is on the SCG domain across 2, 4, 6, and 9 month sampling periods. IRT modeling was used to estimate theta (*θ*), an index of the latent trait of social/communication/cognition, for each infant at each period using the SCG domain items (35, 39). Mean theta values are on a scale similar to a *z*-score; a distribution centered at zero with a standard deviation metric (40). Reliability estimates ranged from .97 to .99 across all time periods and the dimensionality of the items at each sampling period has been established *via* exploratory factor analyses (under review).

Survey questions about sociodemographic characteristics, including maternal age and level of education, were completed during the NB period, with relevant information updated at all subsequent assessments. The degree of neighborhood deprivation was also calculated for all participants using the 2018 Area Deprivation Index (ADI), which is a validated, neighborhood-level composite of 17 education, employment, housing-quality, and poverty variables extracted from the American Community Survey and US Census Survey data (41). The ADI is represented as a state decile ranking score ranging from 1 to 10, with the least resourced neighborhoods, or census block groups, characterized by higher scores and the most resourced by lower scores.

Thirty-two validity items were specifically developed for PediaTrac to help assess response style and ensure that any variabilities in responding, if present, are accounted for in the prediction models. Validity items target three potential sources of distortion: atypical responding (ATP; previously referred to as random or RND), positive (PRS) and negative (NRS) response style. Atypical responding is operationalized as unusual endorsement of statements that have an obvious answer. The logic behind the ATP scale is that all *bona fide* examinees who are literate, proficient in English and attend to item content should be able to choose the one correct option. The NRS scale consists of items that provide an evaluative statement of the infant in the negative direction (i.e., indicating harsh judgment or an overly pessimistic outlook on the child's future). Conversely, the PRS scale consists of items that provide an evaluative statement of the infant in the positive direction (i.e., indicating unrealistically positive opinion or an overly optimistic outlook on the child's future). Caregivers were required to respond to a 5-point Likert scale with response anchors as follows: 1 = never; 2 = rarely; 3 = sometimes; 4 = often; 5 = always. Whether a given validity item endorsement was indicative of non-credible responding was determined based on the frequency of that response in the sample. If <10% of the individuals endorsed the item, it was considered invalid (i.e., strong evidence of non-credible responding). Items were scaled such that higher scores represented increased deviation from a more typical response pattern (42).

The Ages and Stages Questionnaire (ASQ-3) is a caregiver report for children 1–66 months of age and is one of the most widely used developmental caregiver instruments (20). The ASQ-3 is comprised of five scales (Communication, Gross Motor, Fine Motor, Problem Solving, and Personal-Social) as assessed using 21 questionnaires, one for each 2 to 3 month age interval. For the purpose of the current project, only Communication, Problem Solving, and Personal-Social scales were utilized in the analyses given that the content of these scales most logically maps to the SCG domain of PediaTrac. The ASQ-3 provides cut-off scores that designate either no concerns, the need for monitoring, or the need for further assessment. However, for the purposes of the current study, ASQ-3 scores were converted to *z*-scores based on means and standard deviations reported in the administration manual. For infants born preterm, caregivers completed the age-corrected ASQ-3 measure.

### 2.4. Statistical analyses

#### 2.4.1. Preliminary analyses

Descriptive and exploratory analyses were conducted to examine the impact of covariates such as demographic (i.e., caregiver age at study enrollment) and sociodemographic characteristics (i.e., caregiver education, ADI), and social/communication/cognition theta values and ASQ z-scores for Communication, Problem Solving, and Personal-Social scales at the 2, 4, 6, and 9 month sampling periods.

#### 2.4.2. Main analysis

First, to demonstrate convergent validity between the established scales of the ASQ-3 and the PediaTrac SCG domain, Pearson correlation coefficients were computed to examine the relationships between caregiver-reported social/communication/cognitive skills using the PediaTrac SCG domain thetas and *z*-scores for the ASQ-3 scales at each sampling period. This was a necessary first step in order to ensure that the possible effects of sociodemographic and response style could be validly interpreted as impacting related outcomes and caregiver report generally.

Second, and the primary aim of the investigation, to examine the role of sociodemographic variables and caregiver response style on SCG domain theta and ASQ-3 scales, separate cross-sectional linear regression models were conducted at each sampling period. Caregiver response styles (positive, negative or random) were the predictors in the regression models and the SCG domain thetas and z-scores for the ASQ-3 scales were the outcomes. All models were adjusted for sociodemographic characteristics (i.e., caregiver age, infant age, ADI), as well as caregiver race, which was dichotomized given that it was a categorical variable and the nature of the analyses (Black vs. non-Black and White vs. non-White). Infant age in weeks (uncorrected for premature infants) was used in analysis of age effects to obtain a more precise estimate than would be possible using sampling period as a proxy for age. *Post hoc* partial correlations were used to examine whether the relationship between caregiver response style and PediaTrac (SCG domain theta) outcomes were moderated by SES, for linear regression models that showed significant main effects.

## 3. Results

### 3.1. Sample characteristics

The current study sample included 571 caregivers-infant dyads. Caregivers had a mean (standard deviation) age of 30.1 (6.04) years; 53.5% were married; and 76.7% had some college or higher education. Forty-eight percent of the infants were female, 58% were born full-term, and 34% were African American/Black. See Table 1 for descriptive statistics for the full sample.

**Table 1:** Pooled demographics and characteristics of infants and caregivers.

**Infant Biological Sex**	
Male	298 (52.2%)
Female	273 (47.8%)
**Term Status**	
Full-term	331 (58.0%)
Pre-term	240 (42.0%)
**Gestational Age at Birth**	
Full-term	39.0 ± 1.15
Pre-term	33.0 ± 2.96
**Infant Race** *	
African American/Black	192 (34.1%)
Multiracial	58 (10.3%)
Other	5 (0.89%)
White	272 (48.3%)
**Infant Ethnicity** *	
Hispanic/Latino	36 (6.39%)
Caregiver Age at Enrollment (years)	30.1 ± 6.04
Household ADI	5.32 ± 3.33
**Household Income**	
Below poverty	169 (32.8%)
Below median	70 (13.6%)
At/above median	122 (23.7%)
At/above twice median	88 (17.1%)
Above $150,000	66 (12.8%)
**Maternal Education**	
Some/completed high school	133 (23.3%)
Some college or trade, technical, or vocational training	160 (28.0%)
College graduate	136 (23.8%)
Some/completed postgraduate or professional degree	142 (24.9%)
**Caregiver Marital Status**	
Married	305 (53.5%)
Not married	265 (46.5%)

* Self-identified race categories African American or Black, Multiracial, White and Other were non-Hispanic. Race was unknown for 8 infants. 98% of caregivers were biological mothers. Marital status was missing for 1 caregiver. ADI = Area Deprivation Index. Gestational age at birth, maternal age, and household ADI are presented as mean ± standard deviation. Income was categorized relative to the US Department of Health and Human Services Poverty Guidelines (2019) and median household income for Michigan. It should be noted that the difference in median household income in 2019 for Ohio, though smaller, was similar enough to Michigan that the categorization would be the same whether Michigan or Ohio was used. This categorization is based on the number of people in the home as well as household income. The total number of cases differs for some variables due to missing data, reflecting issues such as the failure of some caregivers to provide data on family income (*n* = 56).

Table 2 reports descriptive statistics of the SCG domain theta scores and ASQ-3 domain *z*-scores. Regarding interpretation of the response style scales, the cutoff associated with the top 5% of the distribution (i.e., most deviant scores) for the PRS, NRS, and ATP response styles are ≥7, 5, and 5, respectively. The base rates of “failure” (%) on the PRS, NRS, and ATP scales at these cutoffs was 9.1, 5.8, and 8.0, respectively.

**Table 2:** ASQ-3, SCG theta, and caregiver response styles means and standard deviations.

Variable	*M* (*SD*)
**ASQ-3 Communication**	
2 Month	−0.30 (1.06)
4 Month	−0.31 (1.04)
6 Month	−0.14 (0.99)
9 Month	0.13 (1.13)
**ASQ-3 Personal Social**	
2 Month	−0.16 (1.0)
4 Month	−0.08 (0.96)
6 Month	−0.15 (1.01)
9 Month	−0.24 (1.06)
**ASQ-3 Problem Solving**	
2 Month	−0.33 (1.02)
4 Month	−0.07 (0.97)
6 Month	−0.03 (0.93)
9 Month	−0.25 (1.21)
**SCG Thetas**	
2 Month	0.03 (0.94)
4 Month	0.19 (0.93)
6 Month	0.65 (0.72)
9 Month	0.72 (0.67)
**Positive Response Style (PRS)**	
2 Month	2.03 (2.61)
4 Month	2.04 (2.70)
6 Month	1.92 (2.73)
9 Month	1.88 (2.72)
**Negative Response Style (NRS)**	
2 Month	1.37 (1.83)
4 Month	1.43 (1.77)
6 Month	1.56 (1.95)
9 Month	1.53 (1.99)
**Atypical Responding (ATP)**	
2 Month	1.54 (1.92)
4 Month	1.69 (2.18)
6 Month	1.68 (2.27)
9 Month	1.70 (2.29)

### 3.2. Bivariate Relationship between SCG domain theta and ASQ-3 domain *z*-score

SCG domain theta scores were significantly positively correlated across all sampling periods with *z*-scores for ASQ-3 Communication (*r*s range .41–.53), Personal-Social (*r*s range .33–.44), and Problem Solving (*r*s range .34–.36) scales. See Table 3.

**Table 3:** Pearson correlation coefficients between SCG thetas and ASQ-3 domains at each sampling period .

	ASQ-3 Communication	ASQ-3 Personal-Social	ASQ-3 Problem Solving
*2 Month Sampling Period*	* *	* *	* *
SCG Theta	.53*	.44*	.35*
*4 Month Sampling Period*	* *	* *	* *
SCG Theta	.41*	.39*	.34*
*6 Month Sampling Period*	* *	* *	* *
SCG Theta	.47*	.33*	.34*
*9 Month Sampling Period*	* *	* *	* *
SCG Theta	.48*	.36*	.36*

Note. ASQ-3 refers to The Ages and Stages Questionnaire and SCG refers to PediaTrac Social/Communication/Cognition domain.

* *p *< .001.

### 3.3. Multivariable linear regression models

Separate multivariable linear regression models were run to examine the impact of sociodemographic variables and caregiver response style on SCG domain theta and ASQ-3 scales at each sampling period. All models included maternal education, maternal age, maternal race, infant age, and ADI state rank, in addition to the three caregiver response styles (NRS, PRS, ATP). Overall model results and significant associations are detailed below, full results are in Table 4.

**Table 4:** Results of multivariate linear regression models examining caregiver response styles and sociodemographic factors on SCG and ASQ-3 domains.

	SCG Theta						ASQ-3 Communication						ASQ-3 Personal Social						ASQ-3 Problem Solving					
	*B*	*t*	*p*	95% CI		*R* ^2^	*B*	*t*	*p*	95% CI		*R* ^2^	*B*	*t*	*p*	95% CI		*R* ^2^	*B*	*t*	*p*	95% CI		*R* ^2^
2 Months						0.22						0.046	* *	* *	* *			0.044						0.024
Positive Response Bias	0.08***	5.04	<.001	0.05	0.12		0.04	1.93	0.06	−0.001	0.08		0.04*	2.03	0.04	0.001	0.08		0.02	1.22	0.22	−0.02	0.07	
Negative Response Bias	−0.02	−0.85	0.40	−0.06	0.02		−0.03	−1.00	0.32	−0.08	0.02		−0.03	−1.07	0.28	−0.08	0.02		−0.03	−1.34	0.18	−0.08	0.02	
Atypical Responding	0.02	1.01	0.31	−0.02	0.06		0.01	0.4	0.69	−0.04	0.06		0.03	1.33	0.18	−0.02	0.08		0.02	0.88	0.38	−0.03	0.07	
Maternal Age	−0.02*	−2.2	0.03	−0.03	−0.002		−0.01	−1.55	0.12	−0.03	0.004		−0.01	−0.81	0.42	−0.03	0.01		−0.02*	−2.14	0.03	−0.04	−0.00	
Maternal Education	−0.08	−1.57	0.12	−0.17	0.02		0.06	0.99	0.32	−0.06	0.19		0.04	0.72	0.47	−0.07	0.16		0.07	1.1	0.27	−0.05	0.19	
Maternal Race																								
Black vs. non-Black	0.07	0.51	0.61	−0.2	0.34		0.14	0.8	0.42	−0.21	0.49		0.2	1.18	0.24	−0.13	0.53		0.06	0.33	0.74	−0.28	0.40	
White vs. non-White	−0.23	−1.9	0.06	−0.47	0.01		−0.03	−0.17	0.87	−0.34	0.29		0.06	0.41	0.68	−0.24	0.36		0.04	0.27	0.79	−0.26	0.35	
ADI	0.01	0.41	0.68	−0.02	0.4		0.02	0.87	0.38	−0.02	0.06		0.01	0.3	0.76	−0.03	0.04		−0.01	−0.33	0.74	−0.04	0.03	
Infant Age (weeks)	0.00	−0.22	0.83	0.00	0.00		−0.001	−0.81	0.42	−0.01	0.00		−0.001	−1.51	0.131	−0.00	0.00		0.00	−0.98	0.33	−0.00	0.00	
4 Months						0.17						0.073						0.024						0.021
Positive Response Bias	0.10***	5.81	<.001	0.06	0.13		0.06**	3.16	0.002	0.02	0.10		0.03	1.76	0.08	−0.004	0.07		0.01	0.52	0.60	−0.03	0.05	
Negative Response Bias	−0.03	−1.24	0.22	−0.07	0.02		−0.01	−0.18	0.86	−0.06	0.05		−0.002	−0.09	0.93	−0.05	0.05		0.01	0.33	0.74	−0.04	0.06	
Atypical Responding	−0.02	0.35	0.35	−0.05	0.02		−0.07**	−2.92	0.004	−0.11	−0.02		0.00	0.02	0.98	−0.04	0.04		−0.04	−1.64	0.10	−0.08	0.01	
Maternal Age	−0.01	−0.96	0.34	−0.02	0.01		−0.02	−1.63	0.10	−0.03	0.00		−0.02*	−2.28	0.02	−0.04	−0.003		0.00	0.27	0.79	−0.02	0.02	
Maternal Education	−0.04	−0.82	0.41	−0.14	0.06		0.003	0.05	0.96	−0.12	0.12		0.09	1.51	0.13	−0.03	0.2		0.06	1.00	0.32	−0.06	0.17	
Maternal Race																								
Black vs. non-Black	0.07	0.49	0.62	−0.21	0.35		−0.24	−1.32	0.19	−0.59	0.12		−0.05	−0.29	0.77	−0.38	0.28		−0.24	−1.55	0.12	−0.61	0.07	
White vs. non-White	0.00	−0.01	0.99	−0.25	0.25		−0.27	−1.7	0.09	−0.58	0.042		−0.06	−0.43	0.66	−0.36	0.22		−0.29	−1.9	0.06	−0.58	0.01	
ADI	0.03	1.87	0.06	−0.001	0.06		0.02	1.12	0.26	−0.02	0.06		−0.01	−0.55	0.58	−0.05	0.03		−0.002	−0.1	0.92	−0.04	0.03	
Infant Age (weeks)	0.01	0.62	0.53	−0.01	0.03		−0.01	−1.16	0.25	−0.04	0.01		−0.01	−1.06	0.29	−0.04	0.01		−0.01	−1.21	0.23	−0.04	0.01	
6 Months						0.16						0.08						0.009						0.011
Positive Response Bias	0.07***	5.66	<.001	0.05	0.10		0.02	1.18	0.24	−0.01	0.06		−0.02	−0.94	0.35	−0.06	0.02		0.01	0.54	0.59	−0.03	0.05	
Negative Response Bias	−0.01	−0.73	0.46	−0.04	0.02		0.003	0.12	0.91	−0.03	0.05		−0.01	−0.4	0.69	−0.06	0.04		−0.01	0.61	0.54	−0.03	0.05	
Atypical Responding	−0.01	−0.9	0.46	−0.04	0.02		0.06**	2.83	0.005	0.02	0.11		−0.01	−0.28	0.78	−0.05	0.04		0.01	0.61	0.54	−0.03	0.20	
Maternal Age	−0.01	−0.96	0.34	−0.02	0.01		0.01	0.59	0.55	−0.01	0.02		−0.01	−0.79	0.46	−0.44	0.2		−0.01	−0.74	0.46	−0.02	0.01	
Maternal Education	0.01	0.24	0.81	−0.07	0.09		−0.05	−0.84	0.40	−0.17	0.07		−0.02	−0.37	0.71	−0.11	0.1		0.09	1.52	0.13	−0.02	0.20	
Maternal Race																								
Black vs. non-Black	0.08	0.7	0.48	−0.14	0.30		0.06	0.37	0.71	−0.28	0.40		0.06	0.3	0.76	−0.30	0.41		0.09	0.52	0.60	−0.24	0.41	
White vs. non-White	−0.03	−0.29	0.77	−0.22	0.17		−0.08	−0.52	0.60	−0.38	0.22		−0.12	−0.74	0.46	−0.44	0.2		0.08	0.58	0.56	−0.21	0.38	
ADI	0.03*	2.2	0.03	0.00	0.05		0.02	1.32	0.19	−0.01	0.02		−0.01	−0.74	0.46	−0.05	0.02		−0.01	−0.35	0.72	−0.04	0.03	
Infant Age (weeks)	0.00	−1.73	0.08	−0.00	0.00		0.00	1.21	0.23	−0.01	0.00		0.00	0.51	0.61	−0.00	0.00		0.00	0.83	0.41	−0.00	0.00	
9 Months						0.164						0.17						0.048						0.044
Positive Response Bias	0.06***	5.22	<.001	0.04	0.09		−0.00	−0.17	0.86	−0.05	0.04		−0.00	−0.17	0.87	−0.05	0.04		−0.05	−1.91	0.06	−0.10	0.00	
Negative Response Bias	−0.01	−0.99	0.32	−0.04	0.01		−0.08**	−3.01	0.003	−0.13	−0.03		−0.02	−0.91	0.36	−0.07	0.03		−0.07*	−2.49	0.01	−0.13	−0.02	
Atypical Responding	0.01	0.44	0.66	−0.02	0.03		0.05*	2.25	0.025	0.01	0.10		0.07**	2.76	0.006	0.02	0.12		0.06*	2.15	0.03	0.00	0.12	
Maternal Age	−0.01	−1.67	0.09	−0.02	0.00		−0.01	−0.95	0.34	−0.03	0.01		−0.03**	−2.81	0.005	−0.05	−0.01		−0.03*	−2.2	0.03	−0.05	−0.00	
Maternal Education	−0.01	−0.23	0.82	−0.08	0.06		−0.04	−0.56	0.57	−0.17	0.09		0.07	1.02	0.31	−0.06	0.2		0.06	0.77	0.44	−0.09	0.21	
Maternal Race																								
Black vs. non-Black	0.04	0.36	0.71	−0.17	0.25		0.05	0.24	0.81	−0.33	0.43		0.00	0.01	0.99	−0.38	0.38		0.33	1.46	0.15	−0.12	0.78	
White vs. non-White	0.05	0.48	0.63	−0.15	0.24		−0.38*	−2.21	0.03	−0.73	−0.04		0.10	0.58	0.56	−0.24	0.44		0.35	1.74	0.08	−0.04	0.75	
ADI	0.02	1.73	0.08	−0.00	0.04		0.05*	2.24	0.03	0.01	0.09		0.02	0.84	0.4	−0.02	0.06		−0.00	−0.05	0.96	−0.05	0.05	
Infant Age (weeks)	−0.0005*	−3.21	0.001	−0.0008	−0.0002		−0.00	−1.37	0.17	−0.00	0.00		0.00	−1.21	0.23	−0.00	0.00		0.00	−0.76	0.45	−0.00	0.00	

*Note.* *p < .05, **p < .01, ***p < .001.

#### 3.3.1. Caregiver response styles

NRS and ATP were not significantly associated with the SCG domain thetas at any sampling period. However, at 9 months for ASQ-3 Communication and Problem Solving, overall models indicated that NRS and ATP explained a significant moderate to large proportion of variance, *R*^2^ = .17, *F*(9, 425) = 9.66, *p* < .001, and *R*^2^ = .04, *F*(9, 423) = 2.17, *p* = .02, respectively, with maternal age also explaining a significant proportion of variance in ASQ-3 Problem Solving at 9 months. NRS was significantly negatively associated with ASQ-3 Communication and Problem Solving at 9 months (*b* = −.08, *t* = −.30, *p* = .003 and *b* = −.07, *t* = −2.49, *p* = .01, respectively), indicating that higher negative perceptions of infants were associated with lower reported ASQ-3 communication and problem solving abilities.

In addition, at 9 months, the overall model for ASQ-3 Personal Social indicated that ATP and maternal age explained a moderate significant proportion of variance, *R*^2^ = .05, *F*(9, 424) = 2.4, *p* = .01. As such, at 9 months, ASQ-3 Communication, Personal Social and Problem-Solving *z*-scores were positively significantly associated with ATP (*b* = .05, *t* = 2.25, *b* = .07, *t* = 2.76, and *b* = .06, *t* = 2.15, respectively; all *p*s < .01), indicating that higher random responding was related to higher reported communication, personal-social and problem-solving skills at 9 months. See Table 4 for details.

The overall model for ASQ-3 Communication at 4 months indicated that both ATP and PRS at 4 months explained a significant moderate proportion of variance, *R*^2^ = .07, *F*(9, 472) = 4.11, *p *< .001. Here, ATP was inversely significantly associated with ASQ-3 Communication at 4 months (*b* = −.07, *t* = −2.92, *p* = .004), suggesting that higher random responding was related to lower reported communication at 4 months.

All SCG domain models indicated that PRS explained a significant moderate to large proportion of variance, *R*^2^ range = .16–.22, *p*s < .001. In addition, overall models indicated that PRS explained a small but significant proportion of variance in ASQ-3 Personal-Social model at 2 months, *R*^2^ = .04, *F*(9, 482) = 2.45, *p *= .01, and significant moderate proportion of variance in ASQ-3 communication at 4 months *R*^2^ = .07, *F*(9, 472) = 4.11, *p *< .001. In these models, PRS was significantly positively associated with SCG domain thetas at all sampling periods (*b*s range .06–.10, *p*s < .001), ASQ-3 Personal-Social at 2 months (*b* = .04, *t* = 2.03, *p* = .04) and ASQ-3 Communication at 4 months (*b* = .06, *t* = 3.16, *p *= .002), indicating that higher positive perceptions of their infants were related to higher reported PediaTrac social/communication/cognition abilities and ASQ-3 personal-social and communication skills at 2 and 4 months.

#### 3.3.2. Sociodemographic associations

Caregiver age, along with PRS, explained a significant large proportion of variance in the SCG domain theta models at 2 months, *R*^2^ = .22, *F*(9, 512) = 15.9, *p* < .001. Caregiver age, although significantly associated with ASQ-3 Personal-Social at 4 months, did not explain a significant proportion of variance in the model, *R*^2^ = .02, *F*(9, 471) = 1.32, *p *= .22, but did account for significant moderate proportion of variance, along with ATP, at 9 months, *R*^2^ = .04, *F*(9, 424) = 2.40, *p *= .01. Caregiver age, although significantly associated with ASQ-3 Problem Solving at 2 months, did not explain a significant proportion of variance in the model, *R*^2^ = .02, *F*(9, 480) = 1.32, *p *= .23, but did account for a significant moderate proportion of variance at 9 months, *R*^2^ = .04, *F*(9, 423) = 2.17, *p *= .02.

Caregiver age was significantly inversely associated with SCG domain thetas at the 2 month sampling period, with younger caregivers reporting higher infant social/communication/cognition skills (*b* = −.02, *t* = −2.2, *p* = .03). Caregiver age also significantly inversely correlated with ASQ-3 Personal-Social at 4 and 9 months, with younger caregivers reporting higher ASQ-3 Personal-Social skills (*b* = −.02, *t *= −2.28, *p* = .02 and *b* = −.03, *t *= −2.81, *p* < .001, respectively). Similarly, caregiver age was significantly inversely correlated with ASQ-3 Problem Solving scores at 2 (*b* = -.02, *t* = −2.14, *p *= .03) and 9 (*b* = -.03, *t* = −2.2, *p* = .03) months, with younger caregivers reporting higher infant problem solving abilities at 2 and 9 months of age.

With respect to other sociodemographic factors, results of adjusted multivariable regression models indicated caregiver race, along with ATP and NRS, accounted for a significant large proportion of variance in ASQ-3 Communication scores at 9 months, *R*^2^ = .17, *F*(9, 425) = 9.66, *p *< .001. Here, non-White caregivers reported higher ASQ-3 Communication scores at 9 months compared to White mothers only (*b* = −.38, *t *= −2.21, *p *= .03).

Further, the SCG model at 6 months indicated that ADI, along with PRS explained a significant large proportion of variance, *R*^2^ = .16, *F*(9, 489) = 10.70, *p *< .001, such that higher ADI was positively correlated with SCG domain thetas (*b *= .03, *t *= 2.2, *p *= .03) at 6 months. Similarly, ADI, along with NRS, ATP, and caregiver race, explained a significant and large proportion of variation in ASQ-3 Communication scores at 9 months, *R*^2^ = .17, *F*(9, 425) = 9.66, *p *< .00; with lower negative response bias, higher atypical responding, non-white race, and higher levels of education noted in infants with higher reported ASQ-3 Communication (*b *= .05, *t *= 2.24, *p* = .03) at 9 months.

Finally, preterm birth (i.e., as measured by weeks since date of birth), along with PRS, explained a significant large proportion of variance in SCG theta at 9 months, *R*^2^ = .16, *F*(9, 475) = 10.10, *p *< .001. Preterm birth was related to lower SCG theta values at the 9 month sampling period (*b* = −.0005, *t *= 3.21, *p *= .001) in the adjusted regression model. That is, the more preterm the infant was at testing, the lower their SCG abilities were rated, despite administration of the age-corrected version of the test. Preterm birth was not associated with lower ASQ-3 domains in any sampling period.

#### 3.3.3. Post-hoc analyses

To further explore the significant relationship between SCG domain thetas, PRS and sociodemographic characteristics, post-hoc partial correlations were computed for models with significant main effects from the multivariate linear regression analyses.

At all sampling periods, a significant amount of variance (*r*s range = .30–.35) remained between SCG domain theta and PRS, after accounting for the effects of sociodemographic variables (i.e., caregiver education, ADI, infant age). However, the correlation between PRS and infant age (uncorrected for premature infants) at the 9 month sampling period was not significant when accounting for the main effect of SCG domain theta (partial *r *= −.06, *p* = .15); and PRS and infant age were not correlated when accounting for the relationship between PRS and SCG domain theta.

Significant main effects for caregiver response patterns and sociodemographic characteristics were found for the ASQ-3 Communication domain at 9 months. Therefore, partial correlations were computed to examine the associations of ASQ-3 Communication at 9 months with NRS and ATP, accounting for the main effects of maternal race (White vs. non-White (i.e., Black or African American, multiracial) and ADI. Caregiver race (White vs. non-White) was unrelated to ASQ-3 Communication domain after removing the effects of NRS (partial *r *= −.33, *p* = .11). Similarly, ADI was unrelated to ASQ-3 Communication at 9 months after removing the effects of NRS (partial *r *= .31, *p* = .09). Additionally, maternal race (White vs. non-White) and ADI were unrelated to ASQ-3 Communication after accounting for the effects of ATP at 9 months (partial *r*s = −.31 and.30, *p*s < .10 for maternal race and ADI, respectively). Associations of ASQ-3 Communication with NRS or ATP at 9 months remained significant even after accounting for maternal race or ADI in respective partial correlations.

## 4. Discussion

As expected, convergent validity was established between the Social/Communication/Cognition domain of the recently developed PediaTrac, with theta scores consistently and positively correlated with the derived *z*-scores of the Personal-Social, Communication, and Problem Solving scales of the more widely used and established ASQ-3 at the 4, 6, and 9 month sampling periods. Caregiver response style, maternal age, and level of neighborhood deprivation, accounted for significant variance in outcomes on both measures, demonstrating the importance of inclusion of these variables on newly developed measures, as well as for measures already in widespread use.

While the base rates of caregiver positive, negative or atypical response styles were low in the overall sample, these response tendencies accounted for significant variance in the ASQ-3 and PediaTrac caregiver ratings of infant development. PRS accounted for significant variance in caregiver ratings on the PediaTrac SCG domain at all sampling periods and on one ASQ-3 scale at a single sampling period. In contrast, although NRS and ATP were unrelated to PediaTrac caregiver-reported social/communication/cognitive development, both measures of response style were associated ASQ-3 at one or more sampling periods. Maternal age and area deprivation were also associated with PediaTrac and ASQ-3 scores, such that being a younger caregiver and living in an area with more socioeconomic deprivation were associated with caregiver ratings of *better* infant developmental ability. ASQ-3 Communication scores were also higher for infants of caregivers who identified as non-White compared to those identified as White but only at one sampling period.

Some studies have found that performance-based assessments yield higher rates of developmental problems than caregiver report, suggesting under-identification of these problems based on caregiver report only (43). Prior research has identified at least two factors that can compromise the validity of caregiver report including response style (44) and sociodemographic factors (10, 12, 45). However, to our knowledge no existing caregiver report of early development considers these factors in interpreting caregiver ratings. The findings of this study demonstrate the importance of considering these factors into caregiver report and accounting for them in drawing conclusions about children's actual level of developmental functioning.

Because response style and sociodemographic information are not typically incorporated into screening measures of early infant development, normative reference groups may not provide accurate estimates of an individual child's abilities. The absence of objective metrics on response style may also lead to the misidentification of children and to inaccurate estimated of the base rates of developmental problems in young children. The possibility of misidentification of developmental problems is particularly urgent in view of the fact that caregiver report is typically the only feasible method for formally assessing an infant's developmental status and milestone acquisition as part of pediatric well child visits. Integrating reporter response style and sociodemographic characteristics into the most commonly used assessments is thus well justified as a means for improving the accuracy of these reports. Inclusion of measures of response bias is also consistent with the need for accurate and systematic early childhood developmental surveillance systems (4, 5).

The present study has several noteworthy limitations. First, although caregiver reports were collected longitudinally through 24 months of age, the current investigation focused on cross-sectional findings during early infancy, limiting our ability to examine potential differences in the nature and implications of response styles and sociodemographic factors across a wide span of early childhood development (46). Additionally, the study design of PediaTrac excluded caregivers under 18 years of age, which may have limited our ability to capture the full range of influence of younger caregiver age on caregiver report. Lastly, restriction of the study sample to caregivers with English language proficiency and functional literacy skills may also have restricted our ability to identify the full range of sociodemographic influences on caregiver reporting.

## 5. Future directions

The present study demonstrates the need to take caregiver response styles and sociodemographic factors into account in interpreting caregiver ratings of infant development. Although additional investigation of the effects of these factors on caregiver ratings is planned as part of the larger PediaTrac study, further research is needed to better understand the factors that contribute to variations in response style. A deeper understanding of the reasons for the different ways in which caregivers approach child developmental reporting and an understanding of how persons from various backgrounds approach caregiver questionnaires would allow for more inclusive screening of infants and improve the accuracy of these data.

The development of PediaTrac continues as an ongoing longitudinal, multi-site investigation. To reduce burden and enhance utilization and clinical acceptance, we anticipate designing an adaptive [i.e., computer adaptive test (CAT)] version of PediaTrac that caregivers can complete on an iPad or other digital source that can be integrated into the electronic medical record (EMR) system, and for which trajectories of development can be visualized in real-time. The investigators are currently using a host of data analytic methods to develop complex algorithms for which socio-demographic and response style sources of variation can be corrected or systemically accounted for at the time of assessment so that more precise and individualized estimates of infant or toddler developmental status (e.g., motor, social/communication cognition, sleep, etc.) can be obtained.

## Data Availability

The original contributions presented in the study are included in the article/Supplementary Materials, further inquiries can be directed to the corresponding author.

## On Behalf Of

**PediaTrac Project Consortium

Casey Swick^1^, Samantha Goldstein^1^, Michelle Lobermeier^1^, Amanda Hicks^1^, Natasha Lang^1^, Gabrielle LeDoux^1^, Shannon Franz^2^, Najae Dixon^2^, Kirsten Oard^2^, Katherine Rosenblum^3^, John Barks^4^, Lesa Dieter^5^, Jazmine Kirkland^5^, Yanisa Robbins^5^ and Emily Gorjanc^5^

^1^Psychology, Eastern Michigan University, MI, United States

^2^Physical Medicine and Rehabilitation, University of Michigan, MI, United States

^3^Department of Psychiatry, University of Michigan, MI, United States

^4^Department of Pediatrics, University of Michigan, MI, United States

^5^Pediatrics, Rainbow Babies & Children's Hospital, Case Western Reserve University, OH, United States
